# Biomonitoring via DNA metabarcoding and light microscopy of bee pollen in rainforest transformation landscapes of Sumatra

**DOI:** 10.1186/s12862-022-02004-x

**Published:** 2022-04-26

**Authors:** Carina Carneiro de Melo Moura, Christina A. Setyaningsih, Kevin Li, Miryam Sarah Merk, Sonja Schulze, Rika Raffiudin, Ingo Grass, Hermann Behling, Teja Tscharntke, Catrin Westphal, Oliver Gailing

**Affiliations:** 1 Department of Forest Genetics and Forest Tree Breeding, University of Göttingen, 37077 Göttingen, Germany; 2 Department of Palynology and Climate Dynamics, Albrecht-von-Haller-Institute for Plant Sciences, University of Göttingen, 37073 Göttingen, Germany; 3 Agroecology, Department of Crop Sciences, University of Göttingen, Grisebachstrasse 6, 37077 Göttingen, Germany; 4 Statistics and Econometrics, University of Göttingen, Göttingen, Germany; 5 Department of Biology, IPB University ID, Bogor, West Java 16880 Indonesia; 6 Department of Ecology of Tropical Agricultural Systems, University of Hohenheim, 70599 Stuttgart, Germany; 7 Functional Agrobiodiversity, Department of Crop Sciences, University of Göttingen, Grisebachstrasse 6, 37077 Göttingen, Germany; 8 Centre of Biodiversity and Sustainable Land Use, University of Göttingen, 37077 Göttingen, Germany

**Keywords:** Environmental DNA, Biodiversity, Taxonomic composition

## Abstract

**Background:**

Intense conversion of tropical forests into agricultural systems contributes to habitat loss and the decline of ecosystem functions. Plant-pollinator interactions buffer the process of forest fragmentation, ensuring gene flow across isolated patches of forests by pollen transfer. In this study, we identified the composition of pollen grains stored in pot-pollen of stingless bees, *Tetragonula laeviceps*, via dual-locus DNA metabarcoding (ITS2 and *rbcL*) and light microscopy, and compared the taxonomic coverage of pollen sampled in distinct land-use systems categorized in four levels of management intensity (forest, shrub, rubber, and oil palm) for landscape characterization.

**Results:**

Plant composition differed significantly between DNA metabarcoding and light microscopy. The overlap in the plant families identified via light microscopy and DNA metabarcoding techniques was low and ranged from 22.6 to 27.8%. Taxonomic assignments showed a dominance of pollen from bee-pollinated plants, including oil-bearing crops such as the introduced species *Elaeis guineensis* (Arecaceae) as one of the predominant taxa in the pollen samples across all four land-use types. Native plant families Moraceae, Euphorbiaceae, and Cannabaceae appeared in high proportion in the analyzed pollen material. One-way ANOVA (p > 0.05), PERMANOVA (R² values range from 0.14003 to 0.17684, for all tests p-value > 0.5), and NMDS (stress values ranging from 0.1515 to 0.1859) indicated a lack of differentiation between the species composition and diversity of pollen type in the four distinct land-use types, supporting the influx of pollen from adjacent areas.

**Conclusions:**

Stingless bees collected pollen from a variety of agricultural crops, weeds, and wild plants. Plant composition detected at the family level from the pollen samples likely reflects the plant composition at the landscape level rather than the plot level. In our study, the plant diversity in pollen from colonies installed in land-use systems with distinct levels of forest transformation was highly homogeneous, reflecting a large influx of pollen transported by stingless bees through distinct land-use types. Dual-locus approach applied in metabarcoding studies and visual pollen identification showed great differences in the detection of the plant community, therefore a combination of both methods is recommended for performing biodiversity assessments via pollen identification.

**Supplementary Information:**

The online version contains supplementary material available at 10.1186/s12862-022-02004-x.

## Background

Rainforest composition is highly reliant on pollinators (insects and other groups) to facilitate plant reproductive interactions [1]. Likewise, pollinators rely on flowering plants as a nutritional source [2]. Patterns of pollen dispersal frequently reflect pollinator foraging preferences (e.g. generalist or specialist species) in response to plant mating strategies [3]. These dynamics between plants and pollinators play an important role in establishing community structure and distribution range of species [3]. Bees (Hymenoptera: Anthophila) are considered one of the main pollinators of tropical trees [4], and declines in bee populations have been caused by the reduction of resources due to land-use intensification [2]. Barnes et al. [5] showed that land-use-induced changes in tropical forests alter species richness by direct and cascading effects on landscape conversion, with negative impacts on plant communities and, consequently, on associated pollinators. Several studies pointed out that forest fragmentation and habitat conversion triggered by agricultural intensification promote a decline in plant-pollinator composition and changes in functional diversity [1–5]. In Southeast Asia, large-scale rainforest conversion into monocultures (e.g. oil palm and rubber) is triggering a steep increase in species extinction rate and consequently leading to a loss in ecosystem functioning and services [5, 6]. Disturbances driven by agricultural intensification in interactions between plants and pollinators interrupt functional composition and reduce the diversity of pollinators, consequently harming pollination services [7–9]. However, detailed evidence of the impact of land-use intensification on specific pollinators and their developmental plasticity remains lacking [10].

Pollen availability is a function that can be used to extrapolate measures of community diversity at the landscape level, with more information on the seasonal dynamics of the landscape. The intensification of land-use changes from forest to agricultural systems leads to a ubiquitous decrease in native plant diversity that rebounds on plant-pollinator interactions [5, 11, 12]. Knowledge of pollen composition and plant species diversity allows us to understand functional plant-pollinator interactions, and it might reflect species response to environmental disturbance and biodiversity losses [13]. However, once the plant diversity of habitats adjacent to the hive or nesting sites decreases, the foraging distances of bees increase proportionally in response to the impact of the land-use transformations [14–18]. Thus, the resource gap triggered by the intensification of land use can be buffered by the increase in bees’ foraging distances [14]. And the pollen influx between fragmented landscapes can be a proxy for landscape connectivity, as long-distance gene flow by pollen connects species from isolated patches via pollination[19].

Plant-pollinator interactions are usually investigated based on observation of host-plant visitation or sampling of the pollinators and later identification of pollen carriage through morphological characters, which demands time and expertise [20, 21]. The most used approach to assess the floral composition of pollen is via light microscopy using micro-morphological analysis of pollen. However, this process can be time-consuming and laborious, and the interpretation of results can be challenging since pollen of some species can be difficult to distinguish [21–24]. DNA metabarcoding approaches have emerged as an alternative for biodiversity surveys without prior taxon identification, enabling simultaneous sequencing and multi-taxa identification of mixed material, and among other applications, it is well suited to disentangle plant-pollinator interactions without the requirement for palynological knowledge [21, 25]. High yield sequencing produces a large number of barcode sequences allowing the identification of multiple species in a single reaction but does not have the precision to infer the relative abundance of the pollen types due to possible quantitative biases produced during DNA isolation, amplification, and sequencing [26]. For plant material identification, including pollen grains, universal chloroplast markers (*rbcL* and *matK*) have proven to be reasonably successful [27]. Most of the studies have employed the plastid region *rbcL* together with the nuclear ribosomal marker ITS2 because the selection of plant barcode markers should be a balance between universality and discrimination, this is achieved by employing both *rbcL* and ITS2 regions [28].

Stingless bees (Hymenoptera: Apoidea: Apidae: Meliponini) are recognized as resilient to disturbance, due to their ecological plasticity and capability of long-distance dispersal in agricultural or degraded landscapes [29]. In this study, we selected a generalist species model, the stingless bee species *Tetragonula laeviceps* (Smith, 1857), which is widely distributed through landscape mosaics in Southeast Asia composed of remnants of rainforest, shrub, rubber, and oil palm plantations. Stingless bees deposit the foraged pollen in cerumen pots, or pot-pollen, which contain wax and resin [30]. Identifying pollen stored in pot-pollen can be highly informative to understand bee foraging behavior and for biomonitoring of terrestrial ecosystems. Despite the decline of specialist pollinators because of forest conversion in tropical landscapes, stingless bees and other generalist pollinators play an important role in the maintenance of ecosystem functioning. They offset pollination scarcity of specialist pollinators and indirectly restore pollination functions [29, 31]. Colony fitness, reproduction, and diversity of pollen resources have been associated with plant species richness [10, 32], however, the composition of pollen resources required to maintain colonies of *T. laeviceps* in converted agricultural systems remains unknown. In this study, we installed hives of *T. laeviceps* in converted rainforest areas in central Sumatra, Indonesia, and employed multi-locus DNA metabarcoding and light microscopy (i) to identify the composition of *T. laeviceps* pot-pollen, (ii) to compare the taxonomic assignments of pollen using both techniques, and (iii) to assess resource use applying both methods across varying land use types (forest, shrub, rubber, and oil palm).

## Results

**Fig. 1:**
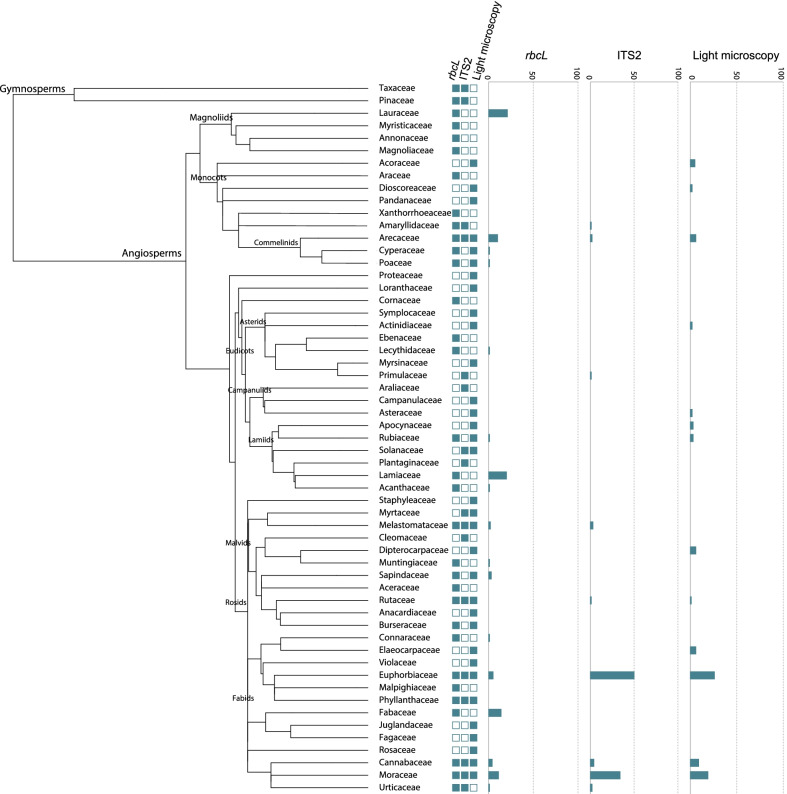
Annotated phylogeny of plant families identified in the pollen samples using dual-locus metabarcoding and light microscopy. Followed by the representation of the plant families detected by metabarcoding loci (*rbcL* and ITS2) and light microscopy: presence (full square)—absence (empty square) of each plant family. And the barplot of the relative abundance of each detected plant family using the two metabarcoding loci (*rbcL* and ITS2) and light microscopy. *Rare plant families with relative abundance values close to zero are not displayed in the barplot representation

**Fig. 2:**
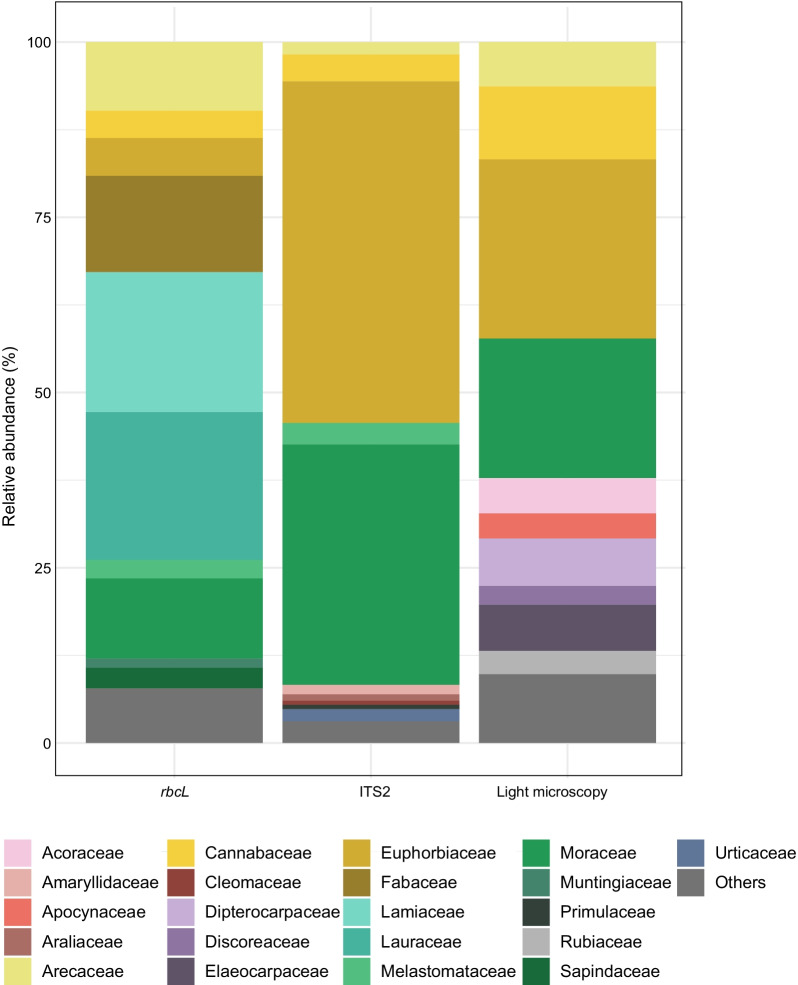
Relative frequency of the ten most abundant plant families detected in pollen samples using dual-locus metabarcoding (*rbcL* and ITS2 markers) and light microscopy. Less abundant plant families were grouped as “Others”

A total of 31 samples of mixed pollen material from 18 plots were successfully analyzed in this study (Additional file 6: Table S1). We detected 72 plant families, 93 genera, and 99 species using both metabarcoding and light microscopy (Additional file 7: Table S2, Additional file 8: Table S3, and Additional file 9:Table S4). After filtering taxa with low coverage (< 1% of reads per sample) in the metabarcoding data sets, 53 families, 63 genera, and 53 plant species were detected using metabarcoding and light microscopy. The plant families Moraceae, Euphorbiaceae, Cannabaceae, and Arecaceae were identified as the most abundant plant families by DNA-based assessment and morphological identification (Figs. 1, 2).

### Sequencing data

The high-throughput sequencing provided a total of 1,746,360 paired-end reads for *rbcL*, ranging from 17,803 to 166,036 reads per sample. The mean number of reads for *rbcL* per sample was 58,212. The total number of merged reads was 1,450,655 for *rbc L,* and a total of 1 ,211,945 paired-end reads with Phred Quality Score (Q) higher than 19 after merging forward and reverse reads. The mean paired-end length of joined reads was 246 bp ranging from 100 to 486 bp.

The total number of pair ed-end reads for ITS2 was 1,760,404, the number of reads varied from 11,274 to 197,501 per sample, with a mean of 56,787 reads per sample. After merging fo rward and reverse reads, we obtained a total of 969,276 reads for ITS2*,* and 833,062 pai red-end reads remained after filtering (Q > 19, sequence length > 100 bp). The sequences presented a mean length of 196 bp and a length ranging from 100 to 533 bp after merging forward and reverse reads for each locus.

After taxonomic annotation of sequences, a total of 559,349 reads of *rbcL* with an average sequence length of 447 bp, and 234,771 reads of ITS2 with an average length of 419 bp were taxonomically assigned. For *rbcL*, more than 84% of all the sequences obtained in this study were assigned with more than 99% of similarity to the sequences in the reference database. While for ITS2, 60% of all the sequences were assigned with more t han 99% of similarity to the reference database (Additional file 1: Fig. S1). Species accumulation curves plotted using species richness and sequence depth obtained for each marker were close to saturation, indicating that our sampling extensively reflects the biodiversity of the studied area (Additional file 1: Fig. S1).

### OTU assignments in dual-locus metabarcoding datasets

OTU assignments based on the dual-locus approach identified a total of 59 families, 97 genera, and 98 plant species (Additional file 7: Table S2, Additional file 8: Table S3). The nuclear ITS2 region distinguished 29 families, 43 genera, and 50 plant species (Additional file 7: Table S2); and a total of 54 families, 78 genera, and 58 plant species were identified using *rbcL* (Additional file 8: Table S3). Redundant taxa identified by both barcode regions represented 40.7% (N = 24) of families (Additional file 2: Fig. S2), 23.5% (N = 23) of genera, and 7.1% (N = 7) of the plant species. The plant composition and total frequency of each plant family detected by ITS2 and *rbcL* were significantly different (Wilcoxon signed-rank test: V = 11,036, p-value < 0.001).

After removing the taxa with low proportion of sequence reads (< 1% reads of each sample), OTU assignments of both loci recovered 32 plant families, 46 genera, and 52 species (Additional file 7: Table S2, Additional file 8: Table S3). Taxonomic assignments using the ITS2 marker recovered 16 families, 19 genera, and 22 species; in contrast, *rbcL* sequences were assigned to 26 families, 36 genera, and 32 species. In total, OTU taxonomic assignments were redundantly identified by both loci after the removal of low sequence readings in the samples (< 1%) in 31.3% (N = 10) of the plant families, 19.6% (N = 9) of the genera, and 3.8% of the species (N = 2). Among the taxa with the highest proportion of reads, Euphorbiaceae and Moraceae appear as dominant plant fami lies detected using ITS2 in respectively 49% and 34% of the reads. Cannabaceae (4%), Melastomataceae (3%), Urticaceae (1.7%), and Arecaceae (1.7%) comprise the next most abundant plant families identified using ITS2 (Figs. 1, 2, Additional file 7: Table S2). In addition, taxon composition detected using *rbcL* revealed that 21% of reads were assigned to Lauraceae and 20% to Lamiaceae. The following most abundant plant families were Fabaceae (14%), Moraceae (11.5%), Arecaceae (10%), and Euphorbiaceae (5.5%) (Figs. 1, 2). Most plants detected by OTU assignments using *rbcL* were classified as native, except for Arecaceae and Muntigiaceae (Additional file 8: Table S3).

### Pollen composition by light microscopy

A total of 42 pollen morphotypes were identified using light microscopy. All morphotypes could be assigned at the family level (N = 33), 20 morphotypes were identified at the genus level, and only 2 morphotypes were identified at the species level (Additional file 9: Table S4). The most abundant plant families detected via light microscopy were Euphorbiaceae (26%), followed by Moraceae (20%), Cannabaceae (10%), Dipterocarpaceae (7%), Elaeocarpaceae (7%), Arecaceae (5%), and Acoraceae (5%) (Figs. 1, 2).

### Comparing dual-locus metabarcoding and light microscopy datasets

Only 19.4% (N = 14) of pla nt families were redundantly detected by ITS2, *rbcL*, and light microscopy (Additional file 2: Fig. S2-A). The proportion of redundant plant families detected by both methods increased to 27.8% (N = 20) when combining data from both loci and comparing it with the plant families detected by light microscopy (Additional file 2: Fig. S2-B). After excluding taxa detected in less than 1% of the total reads per sample, only 11.3% (N = 6) of the plant families were detected by ITS2, *rbcL* and light microscopy (Additional file 2: Fig S2-C). In contrast, a total of 22.6% (N = 12) of plant families were detected by combining the taxonomic assignments obtained using the two metabarcoding loci and light microscopy (Additional file 2: Fig. S2-D).

The taxa composition at the family level and its proportions assigned by ITS2 and light microscopy were significantly different (Wilcoxon signed-rank test: V = 2200, p-value = 0.011). Likewise, the taxonomic assignments implemented using *rbcL* and light microscopy showed significant differences (Wilcoxon signed-rank test: V = 0.377, p-value < 0.001).

Despite the high number of families detected in the pollen samples, only a few plant families represent about 70% of the total number of sequence reads and counts via light microscopy, indicating a predominance of certain taxa in the samples. The most abundant plant families were Moraceae, Euphorbiaceae, Cannabaceae, Arecaceae, Lauraceae, Lamiaceae, Fabaceae, Dipterocarpaceae, and Elaeocarpaceae (Figs. 1, 2).

### Pollen composition across land use types

**Fig. 3:**
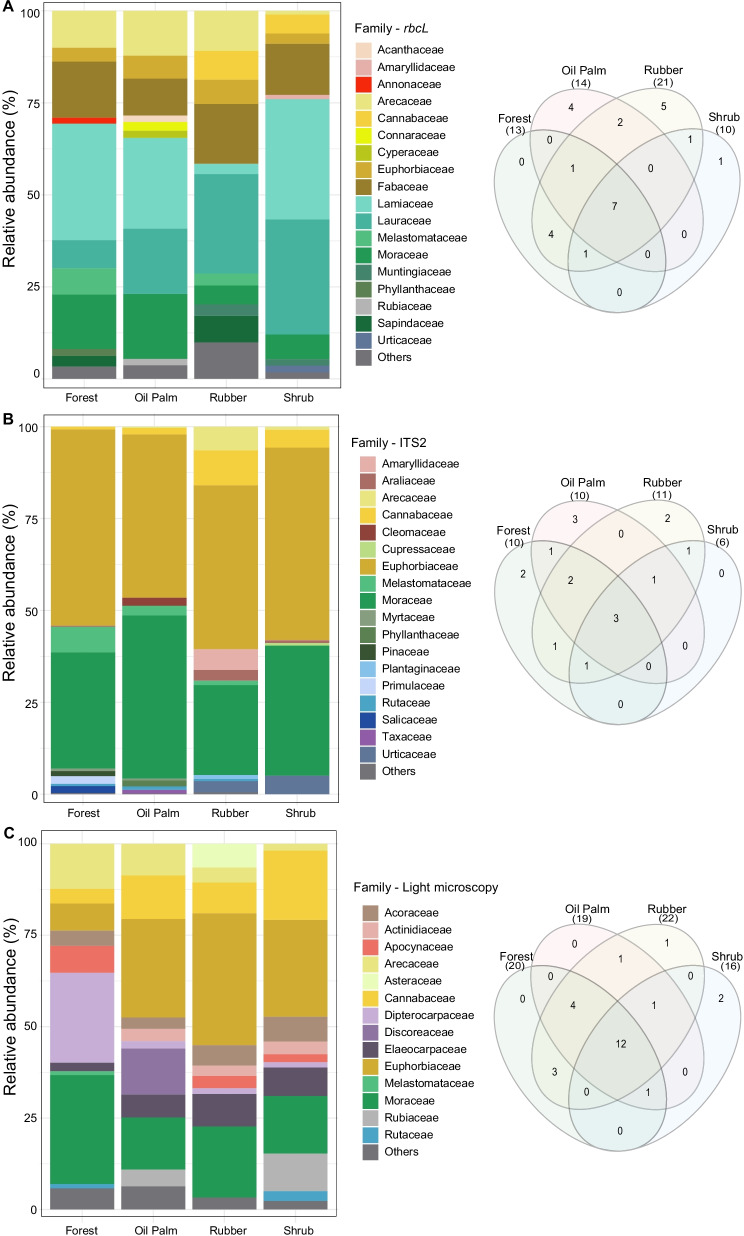
Top 10 most abundant plant families detected per land use type (forest, oil palm, rubber, shrub) by DNA metabarcoding. **A** *rbcL*, **B** ITS2; and **C** light microscopy. On the right, Venn diagrams show the overlap between the occurrence of plant families among the four types of land use

**Fig. 4:**
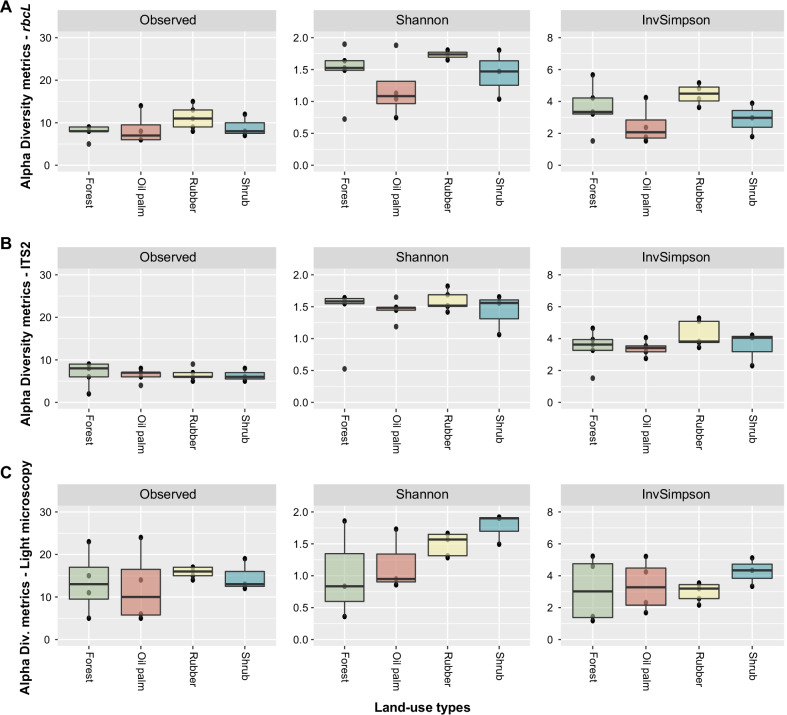
Diversity metrics estimations of plant community detected in pollen samples using OTU assignments obtained using **A** *rbcL*, **B** ITS2 sequences, and **C** and via morphological identification. Colors depict four land use types (forest, oil palm, rubber, shrub). One-way ANOVA of alpha-diversity does not show differentiation between all land use types (p > 0.05) for all methods used in this study

A higher number of plant families were detected in all land use systems using the *rbcL* region compared to ITS2 or light microscopy (Additional file 2: Fig. S2). Kruskal–Wallis tests revealed no significant differences (p-value > 0.05) between the occurrence of individual OTU across the four land use systems for the metabarcoding and light microscopy datasets. NMDS (ITS2 stress value = 0.1859, *rbcL* stress value = 0.1515, and light microscopy stress value = 0.1611, Fig. S3), PERMANOVA (ITS2 R² = 0.14003; *rbcL* R² = 0.17684; light microscopy R² = 0.14705; p-value > 0.5 for all tests; see Additional file 10: Table S5, Additional file 11: Table S6, and Additional file 12: Table S7), and One-way ANOVA (p > 0.05) (Additional file 13: Table S8, Figs. 3, 4) indicated lack differentiation between the pollen composition and pollen diversity in the four land use systems, suggesting that pollen deposited in pot-pollen can be used as a diversity proxy at landscape level rather than reflecting the diversity at plot level.

A wide overlap between the plant families was observed across the four land use types (Fig. 3), however, it is interesting to highlight that pollen from characteristic plant families to primary or secondary forest areas, e.g., Dipterocarpaceae and Phyllanthaceae, were found with a greater proportion in less intensively managed plots (Fig. 3, Additional file 4: Fig. S4). In contrast, pollen from Asteraceae, Cleomaceae, and Urticaceae, which are commonly associated with open habitats, were detected in higher proportions in plots with less percentage of forest cover (i.e. rubb er and oil palm plots), likewise, pollen from agricultural crops, i.e. Discoreaceae, was mostly detected in oil palm plots (Fig. 3, Additional file 4: Fig. S4).

## Discussion

Dual-locus metabarcoding and light microscopy were employed in our study to assess the taxonomic composition of pollen grains from pot-pollen of stingless bees and can be recommended as a landscape biomonitoring tool because it is a time- and cost-effective method for the survey of biological communities [33]. The implementation of dual-locus in metabarcoding studies strikes as a more refined resource for the detection of low abundant taxa in pollen samples [26, 34]. An extensive list of plant families was obtained by the combination of both molecular and morphological-based techniques and lower taxonomic levels could be assessed by the metabarcoding approach. Both methods detected the dominant constituents in the samples and a large number of rare taxa in low proportions. Strikingly, the lack of differentiation in the taxonomic composition and diversity of pollen types sampled in the four land use types (forest, oil palm, rubber, and shrub) was supported by the ANOVA, PERMANOVA, and NMDS analyses, which indir ectly may indicate the influx of pollen from areas adjacent to the monoculture systems.

Taxonomic assignments of mixed pollen using dual-locus metabarcoding and light microscopy showed that the most abundant taxa belong to bee-pollinated plants, including consumable and oil-bearing crops that characterize the study region. Overall, dominant constituents in the samples displayed concordant relative abundances across methods. Moraceae, Euphorbiaceae, Arecaceae, and Cannabaceae were detected with a larger number of counts by both approaches. Other abundant constituents in the samples included the family Melastomataceae with a large number of reads detected by both loci. OTU assigned to the plant families Lamiaceae and Lauraceae were represented in a wide number of reads in the taxonomic assignments using *rbcL* region*,* as well as morphotypes identified as Dipterocarpaceae, Elaocarpaceae, Acoraceae, Apocynaceae, Discoreaceae, and Rubiaceae represented a large proportion of the counts detected by light microscopy.

Variation in the taxonomic coverage by ITS2 and *rbcL* has also been observed in other studies [26, 34]. The variable number of the ribosomal DNA template copies within and between plant species, together with variation in primer binding site-specificity, and uneven DNA concentration of each pollen type could affect the coverage of reads per taxon [35] and explain the lower number of plant families detected in this study for the ITS2 region in comparison with the *rbcL* region. Given that all plant families were available in the reference sequence database used for the taxonomic assignments, the lower number of plant families detected in sequence assignments using the ITS2 dataset was largely linked to the low level of percentage of identity between the query sequences and the reference library. In this study, we employed a threshold of 95% for the percentage of identity as a cut-off for sequence assignments. The sequence similarity score provides information on the taxonomic level, for some taxa, 99% of similarity might provide taxonomic resolution at lower taxonomic levels (e.g. species rank), according to the intraspecific level of polymorphism of the barcode locus and availability of reference sequences. The low percentage of identity between the query sequence and the database sequences for the ITS2 region can be probably linked to the intrinsic characteristics of this ribosomal region, such as problems in amplification due to paralogs and pseudogenes [36], or lower universality and sequence quality compared with plastid regions [37].

In our study, the overlap in the plant families identified with the two techniques ranges from 22.2 to 27.8%. Pollen samples obtained from pot-pollen contain also honey, wax, and other residual material from the colony, and appear to enclose a fairly restricted number of dominant pollen constituents and an extensive number of pollen types in much-reduced proportion, even after deliberate implementation of homogenization steps for each sample. Therefore, multiple subsampling would be essential to access the low abundant taxa present in the samples [23]. Previous studies on pollen metabarcoding revealed that ITS2 failed to identify certain plant families including Lamiaceae and Salicaceae [35]. We found that ITS2 and *rbcL* failed to detect pollen grains from 13 plant families identified by light microscopy (among others Acoraceae, Actinidiaceae, Apocynaceae, Dioscoreaceae, Dipterocarpaceae, and Elaeocarpaceae). DNA obtained from pollen material has proven to be successfully amplified using barcode markers over a large number of species, especially in angiosperms [22, 23, 25, 26, 38]. Pollen grains display a wide variation in morphology (size, shape) of each species, and these morphological traits are most likely expected to play a role in the DNA extraction of the specimens [23]. Distinct outcomes of the taxonomic composition achieved using ITS2 and *rbcL* for pollen identification have also been reported recently [26], and are associated with the differences in the taxonomic coverage of the reference sequence, amplification success, and taxonomic resolution of the two markers [26]. A good compromise for this issue might be to employ a combination of techniques as conducted in the present study.

The percentage of sequence similarity between the query and the reference sequence has a significant impact on the OTU assignment. In our analysis, taxonomic assignments using *rbcL* outperformed ITS2, with considerably higher sequence similarity. This is probably because of the lower level of polymorphism observed in the *rbcL* locus in comparison with the ITS2 region, and it is largely representated in the reference databases. Consequently, higher sequence similarity is therefore expected for *rbcL* compared to ITS2. While similarity scores and phylogenetic relatedness are largely correlated, composition bias and rare heterogeneity reduce this relationship and may cause spurious identification [39]. RDP classifier, a machine learning approach, has been successfully used for plant taxa detection as already shown [26, 34, 40], and the accuracy of taxonomic assignments could be confirmed in our study by verifying the Lowest Common Ancestor (LCA) with the highest sequence similarity score, and other strategies such as strict filtering in the pre-processing data analysis (e.g. removal of singletons and low abundant taxa). Other studies on Sumatra flora faced challenges for the taxonomic identification of some specific clades at the species level [27, 41, 42], which has been associated in some cases with incomplete lineage sorting, hybridization, or low levels of polymorphism of the markers, hindering the OTU assignments at lower taxonomic levels. In this study, 16 OTU assigned to Arecaceae, Burseraceae, Cyperaceae, Fabaceae, Icacinaceae, Lauraceae, Monimiaceae, Poaceae, Rubiaceae, and Rosaceae could only be identified at the family level in the *rbcL* dataset. For the ITS2 sequences, all the OTU could be assigned at the genus level.

The floral composition detected in our study via metabarcoding and light microscopy largely overlays with the most reported interactions between stingless bees and bee-pollinated plants in Indo-Malayan-Australasia [32, 43, 44]. Stingless bees show foraging preferences for flowering plants belonging to the families Fabaceae, Asteraceae, Malvaceae, Euphorbiaceae, Rubiaceae, Arecaceae, and Lamiaceae, spanning a wide variation in form types and covering pollination of crops, native and non-native flora [43, 45, 46]. This reflects the key role of stingless bees in ecosystem functioning and services. In addition, some of these flowering plant families display traits that facilitate pollination via entomophily, as the development of open corollas with numerous stamens allows easy accessibility of pollen and nectar [47] or development of floral resin (e.g. Dipterocarpaceae, Euphorbiaceae, Myrtaceae) that is attractive for resin collecting bees, such as stingless bees [32, 48, 49]. Many of the plants from the above-mentioned families are also known to have evolved as generalists themselves toward pollinators. In order to maintain a polyfloral pollen diet and a large resin diversity, bees face wider foraging distances [32]. Stingless bees from the genus *Tetragonula* have been recorded to forage distances up to 700 m from their nests [50]. The energy cost of large bee movements is rewarded in the form of nutritional content and protection against antagonists achieved through increased resin diversity, which provides several antimicrobial activities and can repel larger predators (i.e., ants) [32]. Our intensively managed study sites located within oil palm and rubber plantations displayed patches of natural habitats within a 500 m radius, this heterogeneity supports the wide floral composition of detected pollen demonstrating environment interlinkages.

The rather homogeneous floral composition of pollen from pot-pollen collected from heterogeneous sites, both lowland forests, shrubland, and agro-ecosystems (rubber and oil palm plantations) offered information on stingless bees foraging behavior and reflects the floristic composition of the landscape. Of particular note is the wide variety of plant families in the pollen obtained from monocultures, where both native and alien plant taxa of different life forms were recorded by DNA metabarcoding and light microscopy. No dramatic shift in biodiversity could be detected among the distinct land use types by analyzing pollen material in this study. This supports the fact that stingless bees are generalist species and actively keep fragmented landscapes sturdily connected via pollen influx from a wide diversity of plant species. It reinforces that intensively managed systems are not essentially nutritional deserts for generalist species, such as stingless bees, and other bee species [35] because bees enhance pollen diversity by foraging in more diverse habitats as a strategy for resource “diversity maximization” [32]. Adjacent areas to field crop agroecosystems uphold a large influx of pollen diversity into converted systems.

Forest conversion to agricultural production promotes direct and cascading impacts on biodiversity [5]. Paradoxically, crop yield is affected by the decline of biodiversity-related ecosystem services [51]. Despite the lack of differentiation in pollen composition identified in the four land use types in our study, higher temperatures have been recorded in more altered land use systems [52]. Higher temperatures and increased exposure is linked to an increased risk of colony mortality [53]. In this context, restoration efforts should be directed to the conservation of remaining forest patches and biological corridors, providing nutritional and nesting resources (such as resins) for pollinators [51]. Stingless bees may be beneficial for counteracting land use fragmentation by supplying pollination services for sustainable agricultural development and conservation of natural ecosystems [54].

Although pollen identification can be applied indubitably as a biomonitoring tool for biodiversity characterization, both light microscopy and DNA metabarcoding methods have major drawbacks. Some plants are difficult to morphologically distinguish at the species level, so unambiguous attribution of species based on sequences available in the reference database is not always possible. Furthermore, reference databases contain sequences from samples with unclear morphological species assignments. This becomes more challenging when pollen originates from tropical species, as the availability of reference vouchers and specialists is scarcer. In addition, the use of local databases for taxonomic assignments provides an equal or higher percentage of plant species detections compared to regional databases and is associated with a lower level of mismatches in OTU assignments [55]. As many tropical species are still lacking reference sequences, in our study we used regional databases for our taxonomic assignments and confirmed the assignments by verifying the Lowest Common Ancestor (LCA) in the distance tree of results. This approach has previously provided high levels of accuracy in taxonomic assignments [56]. Even though quantitative data obtained using metabarcoding has been considered remarkably reliable, variations in DNA extraction efficiency caused by morphological differences of interspecific pollen grains may bias the amplification of the DNA copies and consequently affect the sequencing yield [21, 25]. Cross-contamination of samples is also a known limitation of the metabarcoding approach [25, 26], this problem is often tackled by conservative filtering thresholds, elimination of sequence reads that might appear in the negative control samples, or removal of low abundant taxa per sample (< 1%) [26, 35]. Furthermore, false contaminations produced by tag jumps cause improper sequence assignments to samples and might take place at low proportions and mistakenly inflate diversity [57]. On top of that, a lack of standardization of metabarcoding bioinformatics pipelines represents a challenge for establishing this method in new research groups. Nevertheless, research focusing on optimizing the metabarcoding technique is progressing at leap steps [25, 26, 35, 58–60], as it enables the investigation of new research avenues in plant-pollinator interactions and landscape monitoring.

## Conclusion

Our findings demonstrate that the pollen collection of the generalist bee species *T. laeviceps* is not limited by land use type, and therefore could play an important role in the pollination of wild plants and crops in a heterogeneous landscapes. Fu rthermore, our results point to the application of stingless bees as a successful model for landscape characterization and effective implementation as a large-scale sampling tool via DNA metabarcoding of pollen collected from pot-pollen. Combining light microscopy and dual-locus metabarcoding for pollen identification enables a more refined detection of the floral composition, and it optimizes wildlife monitoring in terms of minimum invasive sampling, and high cost and time efficiency. Both techniques complement each other when applied in tropical studies, since some rare taxa may be difficult to identify by implementing either metabarcoding or light microscopy alone.

## Methods

We characterized the land cover by estimating the proportion of natural forest surrounding the beehives based on manually classified 1.5 m resolution SPOT satellite imagery with the scale of 1:5000 in the program QGIS [61]. The quantified land cover was then compared with supporting imagery in Google Maps and confirmed by field surveys and local expert knowledge (Darras et al. in prep.). We estimated the total percentage of the forest cover using the package “landscapemetrics” [62] in the R version 4.0.3 [63] to determine the fraction of forest and shrub cover within a 500 m radius of each installed hive. We installed three beehives of *T. laeviceps* in 40 plots with coverage of 30 to 70% of forest and shrub vegetation within a 500 m radius set in an agricultural landscape mosaic in Jambi Province, central Sumatra, Indonesia. Sites were designated based on a similar gradient of natural habitat (forest and shrub) composition for all the land use types while maximizing their extremes, this means sites were subjected to the trade-off between oil palm and forest or shrub. Beehives were displayed in boxes under a shelter protected from sun and rain and exposed from July to December 2018 (end of field campaign). Samples of mixed pollen, resin, and wax were collected at the end of the field campaign and maintained frozen at − 18 °C. The pollen composition present in pot-pollen was characterized using DNA metabarcoding and palynological analysis for a total of 31 colonies located in 18 plots (5 plots located in forest, 5 in oil palm, 5 in rubber, and 3 in shrub plots), the remaining hives died or were destroyed (Additional file 5: Fig. S5).

### Pollen identification via light microscopy

Aliquots of 3 mL of mixed pollen, wax, and honey were treated following the standard protocol of the International Honey Commission including acetolysis [64, 65]. One tablet of *Lycopodium clavatum* was added to each sample to estimate palynomorph concentrations [66]. Residues were mounted in glycerol jelly for pollen visualization, identification, and counting. Pollen and spore analyses were carried out using light microscopy. All identified pollen and spore types were photographed using a Leica photomicroscope with a 400 × magnification. Pollen and spores were identified using the tropical pollen reference collections of the Department of Palynology and Climate Dynamics at the University of Göttingen. Pollen was counted and identified up to a total sum of 300 pollen grains per sample on two different slides to maximize randomness.

### DNA extraction, amplification, and high throughput sequencing

Aliquots of 0.5 ml of the samples were washed to remove the remaining beeswax and honey, before DNA extraction by two steps of centrifugation at 11 rpm for 1 min using 1000 µL nuclease-free water and discarding the supernatant and repeating the process two times with 1000 µL ethanol 99%, and a final washing step using nuclease-free water. The samples were transferred to InnuSPEED Lysis Tubes Z (Analytik Jena AG) containing steel and glass mini beads. 400 µL of Lysis Solution CBV was added, and the pollen grains were ruptured using SpeedMill plus (Analytik Jena AG) for four cycles of 4 min each and intervals of 4 min between cycles. DNA extraction was carried out using the Innuprep Plant DNA Kit (Analytik Jena AG), following the manufacturer’s guide.

The barcoding markers *rbcL* and ITS2 were amplified using the primers set rbcL2 [67] and rbcLa-R [68], and ITS2 S2F and ITS2 S3R [69], which yielded PCR products of 350 to 500 bp (Table 1). Each pollen sample was amplified in PCR triplicates/primer, as increasing the number of PCR replicates enhances the number of detected species in metabarcoding samples [70]. PCR reactions contained a final volume of 15.5 µL using 0.2 µL of Taq Hot FirePol (5 U/µL) from Solis BioDyne (Estonia), 1.5 µL of 10X PCR Buffer (with 0.8 M Tris–HCl, 0.2 M (NH_4_)2SO_4_), 1.5 µL of MgCl_2_ (25 mM), 1.5 µL dNTPs (2.5 mM of each dNTP), 6.8 µL H_2_O, 1.5 µL of each forward and reverse primers (5 pmol/µL) and 1 µL DNA (10–20 ng/µL). Thermal cyclic conditions included an initial activation step of 95 °C for 15 min, followed by 35 cycles of 94 °C for 1 min, 50 °C for 1 min, 72 °C for 1 min, and a final extension step of 72 °C for 20 min. Additionally, PCR negative, and positive controls (high-quality DNA from plant material that was successfully sequenced previously) were included in all reactions. PCR cleaning was done using GENECLEAN Kit (MP Biomedicals).

**Table 1:** Barcoding regions, PCR primer sequences, and amplicon sizes

Barcode regions	Primer	Sequence	Size	References
*rbcL*	rbcL 2_f	TGGCAGCATTYCGAGTAACTC	500 bp	Palmieri et al. [68]
	rbcLa-R_r	GTAAAATCAAGTCCACCRCG		Kress and Erickson [69]
ITS2	ITS2 S2F_f	ATGCGATACTTGGTGTGAAT	350–400 bp	Chen et al. [70]
	ITS2 S3R_r	GACGCTTCTCCAGACTACAAT		

We pooled the PCR products of both amplicons before library preparation with a final concentration of 200 ng per sample. Amplicon concentrations were measured using a Qubit fluorescence spectrophotometer (Life Technologies). Dual-index sequencing libraries were prepared using the Illumina TruSeq Nano DNA High Throughput Library Prep Kit (96 samples), and Illumina TruSeq DNA CD Indexes (96 indexes, 96 samples), which ligates A-base ends to the DNA after a phosphorylation step. This step prepares the DNA for ligation to the index sequences and allows the sequence recovery from each sample in the bioinformatics analysis. DNA libraries were loaded at 10 pM concentrations with 10% PhiX control spike and sequenced in one single run carried out on Illumina MiSeq using the MiSeq Reagent Kit v2—300 cycles.

### Data analysis

#### Bioinformatics pipeline

The quality of Illumina raw reads was verified using FastQC [71], followed by the removal of primer sequences and adapters using Cutadapt [72]. Forward and reverse reads were merged using the command -fastq_mergepairs, and singletons, low-quality reads, and short sequences (< Q score 20, < 100 bp, ambiguous base-pairs) were removed with Usearch 11.0.667 [73]. Sequences were dereplicated, sorted by size, and clustered using UPARSE-OTU algorithm in Usearch v. 11.0.667 [74, 75]. Taxonomic assignments of OTU were done using the trained databases available for the regions ITS2 [34] and *rbcL* [76] prepared with the RDP classifier, a machine learning approach based on the naïve Bayes method [40] using 95% identity. Additionally, all OTU sequences were subjected to a BLAST search assessment using blastn in the NCBI Genbank to confirm the accuracy of the taxonomic assignments by verifying the Lowest Common Ancestor (LCA) in the distance tree of results with the highest sequence similarity score. A detailed script of the workflow was added to https://github.com/CarisMoura/Pollen_Metabarcoding_Indonesia-/blob/main/Pipeline.

We plot a histogram of the percentage of similarity score for *rbcL* and ITS2 sequence reads compared against the reference sequence databases used in the study. Accumulation curves of taxa detected in the four land use types (forest, oil palm, rubber, and shrub) were plotted using both metabarcoding loci to evaluate the sample coverage using RStudio, R version 4.0.3 [63].

### Pollen composition by DNA metabarcoding and light microscopy

To reduce possible bias connected to unequal sequencing depth, we opted for conducting all downstream data analysis with taxa more abundant than 1% per sample [35, 77] and normalized the out tables based on mean sequencing depth using the phyloseq package [78] in the R version 4.0.3 [63].

The plant community profile was displayed in a phylogenetic tree and bar plots showing the relative abundance of each taxon assigned to the family level using the sequencing and light microscopy results implemented in TimeTree [79] and annotated using iTOL [80]. Differences in the detected pollen composition using ITS2 and *rbcL* markers, and light microscopy were tested using the Wilcoxon rank-sum exact test implemented in R version 4.0.3 [63]. To facilitate the visualization of the overlap in the detected plant community using the different methods, we plotted Venn’s diagrams of the plant families detected using each approach [81] and bar plots of the relative frequency of the ten most abundant plant families detected per approach using the phyloseq package [78] in the R version 4.0.3 [63].

Kruskal–Wallis test was also implemented for comparisons of individual OTU composition across the four land use types for metabarcoding and light microscopy in R version 4.0.3 [63].

### Pollen composition across land use types

The top 10 most abundant taxa per land use type were displayed in bar plots showing the relative abundance of each taxon assigned to families based on the sequencing and light microscopy results using the R package phyloseq [78]. We inferred alpha diversity (Observed richness, Shannon and InvSimpson index) per land use type (forest, shrub, rubber, and oil palm) using the normalized coun ts of reads in the R package phyloseq with the function estimate_richness [78]. Normality and homoscedasticity of the alpha diversity values were tested using Shapiro–Wilk and Levene’s test (Additional file 14: Table S9), respectively. OTU tables obtained using *rbcL* and ITS2 markers were merged using the function merge_phyloseq in the R package phyloseq. Differences between observed richness detected by ITS2, *rbcL*, and light microscopy across the four land use types were estimated using One-way ANOVA using the function aov in the R package agricolae. Non-metric multidimensional scaling (NMDS) ordination of plant family composition in pot-pollen detected by both DNA-based and morphological approaches was estimated based on the Bray–Curtis dissimilarity between pollen composition of each colony in the four land use types using the R package vegan [82]. We conducted a Permutational Multivariate Analysis of Variance (PERMANOVA) test to estimate dissimilarities in species composition in the different land use types with the function adonis (n = 999 permutations) based on Bray–Curtis dissimilarity.

## Supplementary Information

**Additional file 1: Figure S1. **Percentage of similarity score of *rbcL* and ITS2 sequence reads obtained from mixed pollen samples compared against the taxonomic reference database (at left). Accumulation curve of taxa detected in four land-use types (forest, shrub, rubber and oil palm) using the taxonomic assignments achieved using sequence reads of *rbcL *and ITS2 of pollen material (in the center). Accumulation curve of species richness detected in colonies located at each plot (at right).

**Additional file 2: Figure S2.** Diagrams illustrating overlap between the plant families’ composition detected by dual loci metabarcoding (*rbcL* and ITS2) and light microscopy in pot-pollen samples. A) Total number and percentage of plant families detected using *rbcL*, ITS2, and light microscopy. B) Total number and percentage of families detected by the combined two metabarcoding loci in comparison with the light microscopy results. C) Total number and percentage of families detected using* rbcL*, ITS2 (excluding taxa present in less than 1% of the total number of reads per sample), and palynology. D) Total number and percentage of families detected by the combined two metabarcoding loci (excluding low abundant taxa detected in less than 1% of the total number of reads per sample) in comparison with the light microscopy results.

**Additional file 3: Figure S3.** Non-metric multidimensional scaling of plant family composition in pot-pollen from four land-use types calculated using a Bray-Curtis based on: (A) ITS2 (stress value = 0.1859), (B) *rbcL *(stress value = 0.1515) and (C) light microscopy (stress value = 0.1611). Each point represents the composition of pollen of each plot site located in the four land-use types (forest, oil palm, rubber and shrub).

**Additional file 4: Figure S4.** Top 10 plant families detected in pollen samples via (A) DNA metabarcoding –* rbcL* and (B) ITS2; and (C) light microscopy.

**Additional file 5: Figure S5.** Workflow of experiment design, laboratory experiments, and summary of pollen metabarcoding pipeline.

**Additional file 6: Table S1. **Sample information of pot-pollen material collected from hives installed in four land-use types (forest, shrub, rubber, and oil palm), including sample IDs used in this study, respective colony IDs, plot coordinates, and percentage of natural cover (pcNatural) within a 500 m of each installed hive.

**Additional file 7: Table S2.** OTU assignments and reads count of pot-pollen samples based on the locus ITS2.

**Additional file 8: Table S3.** OTU assignments and reads count of pot-pollen samples based on the locus *rbcL*.

**Additional file 9: Table S4.** Morphological identification of pot-pollen samples based on light microscopy.

**Additional file 10: Table S5. **Permutational Multivariate Analysis of Variance (PERMANOVA) test based on *rbcL* data set with the function Adonis (999 permutations) of the Bray-Curtis dissimilarities.

**Additional file 11: Table S6. **Permutational Multivariate Analysis of Variance (PERMANOVA) test based on ITS2 data set with the function Adonis (999 permutations) of the Bray-Curtis dissimilarities.

**Additional file 12: Table S7. **Permutational Multivariate Analysis of Variance (PERMANOVA) test based on light microscopy data set with the function Adonis (999 permutations) of the Bray-Curtis dissimilarities.

**Additional file 13: Table S8. **Results from One-way ANOVA for the observed richness across the four land-use types (forest, oil palm, shrub, and rubber) detected by ITS2, *rbcL*, both loci merged and light microscopy.

**Additional file 14: Table S9. **Results of Normality test (Shapiro-Wilk) and Homoscedasticity (Levene’s Test) for alpha-diversity metrics (Observed Richness, Shannon and InvSimpson) estimated for ITS2, *rbcL*, the merged loci and light microscopy**.**

## Data Availability

All datasets generated or analyzed during this study are available in the Figshare repository, https://figshare.com/s/789797c51e102383e21c, https://doi.org/10.6084/m9.figshare.19224828.
