# Supplementary material for: Biomonitoring via DNA metabarcoding and light microscopy of bee pollen in rainforest transformation landscapes of Sumatra
Source: BMC Ecol Evol. 2022 Apr 26;22:51. doi: 10.1186/s12862-022-02004-x (PMC9040256; doi:10.1186/s12862-022-02004-x)
Supplement: Supplementary file 1 — Additional file 1: Figure S1. Percentage of similarity score of rbcL and ITS2 sequence reads obtained from mixed pollen samples compared against the taxonomic reference database (at left). Accumulation curve of taxa detected in four land-use types (forest, shrub, rubber and oil palm) using the taxonomic assignments achieved using sequence reads of rbcL and ITS2 of pollen material (in the center). Accumulation curve of species richness detected in colonies located at each plot (at right). [file 12862_2022_2004_MOESM1_ESM.pdf]

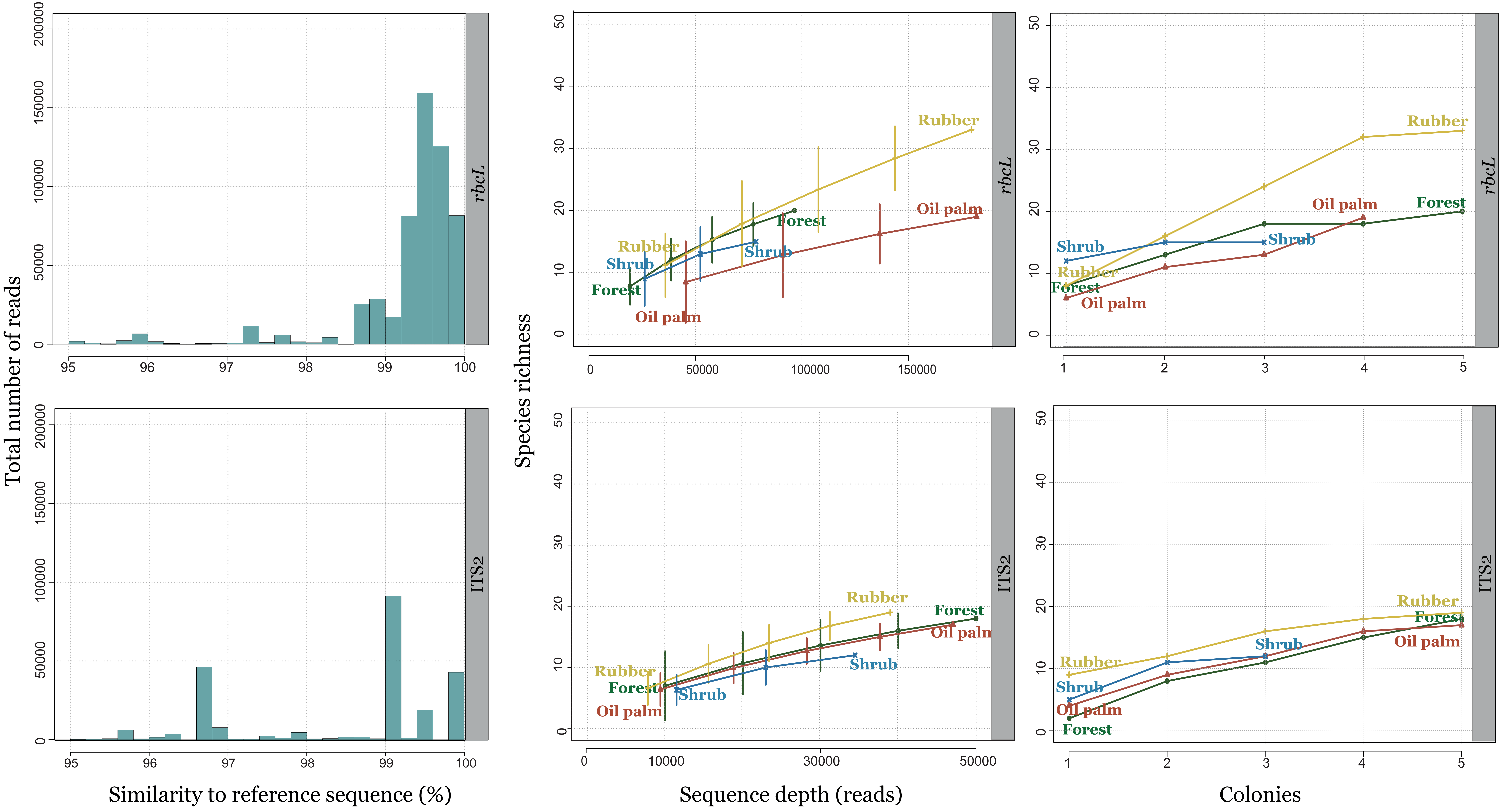

Figure S1. Percentage of similarity score of rbcL and ITS2 sequence reads obtained from mixed pollen samples compared against the taxonomic reference database (at left). Accumulation curve of taxa detected in four land-use types (forest, shrub, rubber and oil palm) using the taxonomic assignments achieved using sequence reads of rbcL and ITS2 of pollen material (in the center). Accumulation curve of species richness detected in colonies located at each plot (at right).
