# Supplementary material for: Biomonitoring via DNA metabarcoding and light microscopy of bee pollen in rainforest transformation landscapes of Sumatra
Source: BMC Ecol Evol. 2022 Apr 26;22:51. doi: 10.1186/s12862-022-02004-x (PMC9040256; doi:10.1186/s12862-022-02004-x)
Supplement: Supplementary file 2 — Additional file 2: Figure S2. Diagrams illustrating overlap between the plant families’ composition detected by dual loci metabarcoding (rbcL and ITS2) and light microscopy in pot-pollen samples. A) Total number and percentage of plant families detected using rbcL, ITS2, and light microscopy. B) Total number and percentage of families detected by the combined two metabarcoding loci in comparison with the light microscopy results. C) Total number and percentage of families detected using rbcL, ITS2 (excluding taxa present in less than 1% of the total number of reads per sample), and palynology. D) Total number and percentage of families detected by the combined two metabarcoding loci (excluding low abundant taxa detected in less than 1% of the total number of reads per sample) in comparison with the light microscopy results. [file 12862_2022_2004_MOESM2_ESM.pdf]

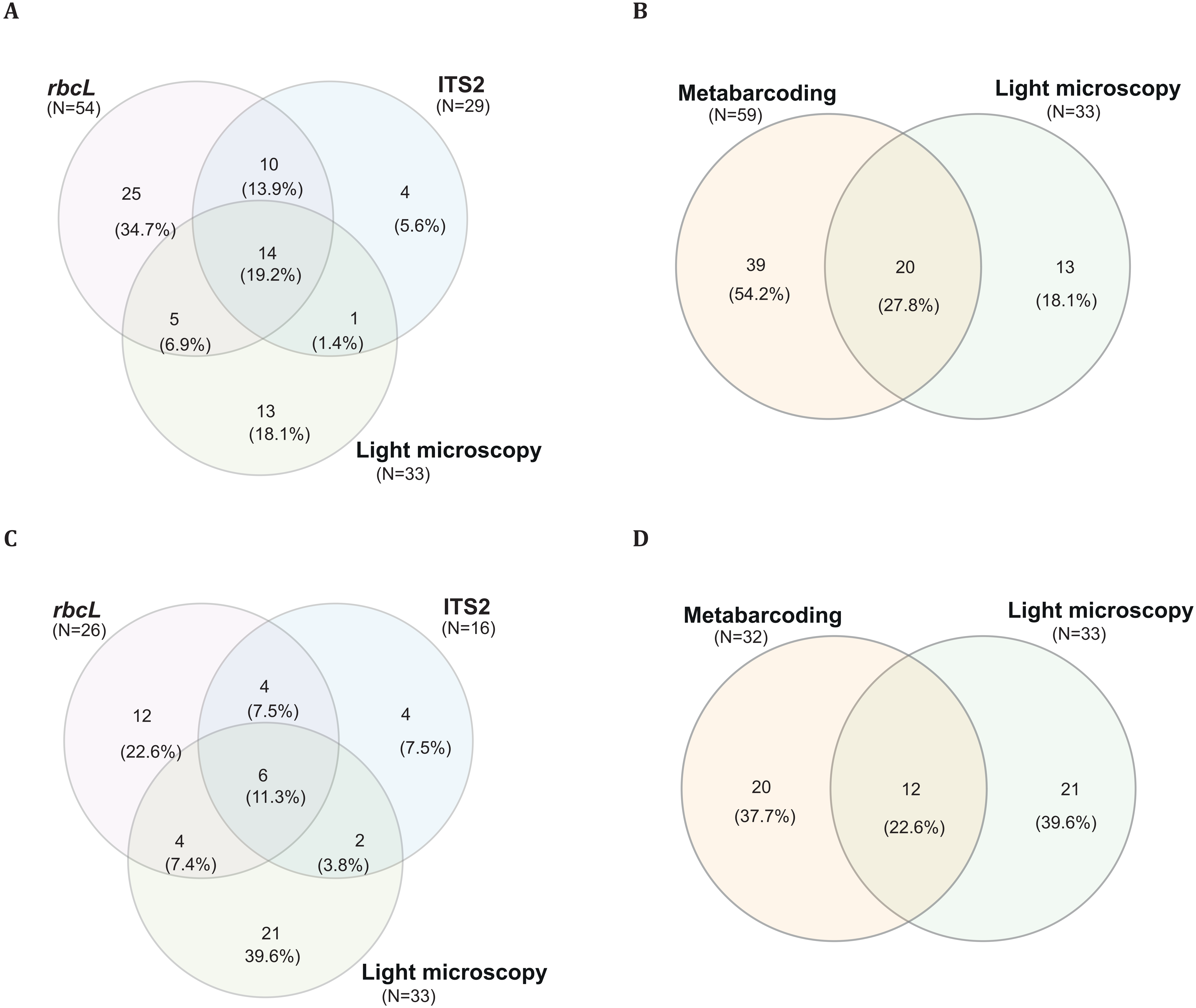

Figure S2. Diagrams illustrating overlap between the plant families' composition detected by dual loci metabarcoding (rbcL and ITS2) and light microscopy in pot-pollen samples. A) Total number and percentage of plant families detected using rbcL, ITS2, and light microscopy. B) Total number and percentage of families detected by the combined two metabarcoding loci in comparison with the light microscopy results. C) Total number and percentage of families detected using rbcL, ITS2 (excluding taxa present in less than 1% of the total number of reads per sample), and palynology. D) Total number and percentage of families detected by the combined two metabarcoding loci (excluding low abundant taxa detected in less than 1% of the total number of reads per sample) in comparison with the light microscopy results.
