# Supplementary material for: Biomonitoring via DNA metabarcoding and light microscopy of bee pollen in rainforest transformation landscapes of Sumatra
Source: BMC Ecol Evol. 2022 Apr 26;22:51. doi: 10.1186/s12862-022-02004-x (PMC9040256; doi:10.1186/s12862-022-02004-x)
Supplement: Supplementary file 3 — Additional file 3: Figure S3. Non-metric multidimensional scaling of plant family composition in pot-pollen from four land-use types calculated using a Bray-Curtis based on: (A) ITS2 (stress value = 0.1859), (B) rbcL (stress value = 0.1515) and (C) light microscopy (stress value = 0.1611). Each point represents the composition of pollen of each plot site located in the four land-use types (forest, oil palm, rubber and shrub). [file 12862_2022_2004_MOESM3_ESM.pdf]

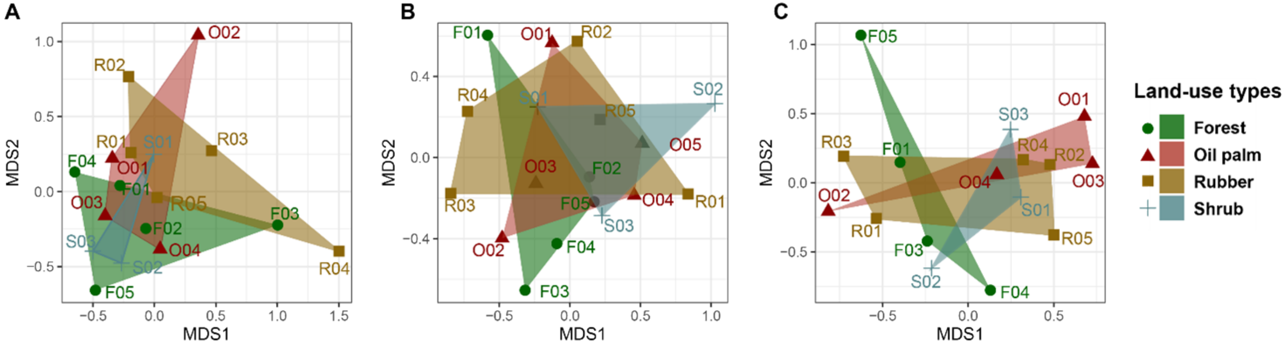

Figure S3. Non-metric multidimensional scaling of plant family composition in pot-pollen from four land-use types calculated using a Bray-Curtis based on: (A) ITS2 (stress value = 0.1859), (B) rbcL (stress value = 0.1515) and (C) light microscopy (stress value = 0.1611). Each point represents the composition of pollen of each plot site located in the four land-use types (forest, oil palm, rubber and shrub).
