# Supplementary material for: Biomonitoring via DNA metabarcoding and light microscopy of bee pollen in rainforest transformation landscapes of Sumatra
Source: BMC Ecol Evol. 2022 Apr 26;22:51. doi: 10.1186/s12862-022-02004-x (PMC9040256; doi:10.1186/s12862-022-02004-x)
Supplement: Supplementary file 4 — Additional file 4: Figure S4. Top 10 plant families detected in pollen samples via (A) DNA metabarcoding – rbcL and (B) ITS2; and (C) light microscopy. [file 12862_2022_2004_MOESM4_ESM.pdf]

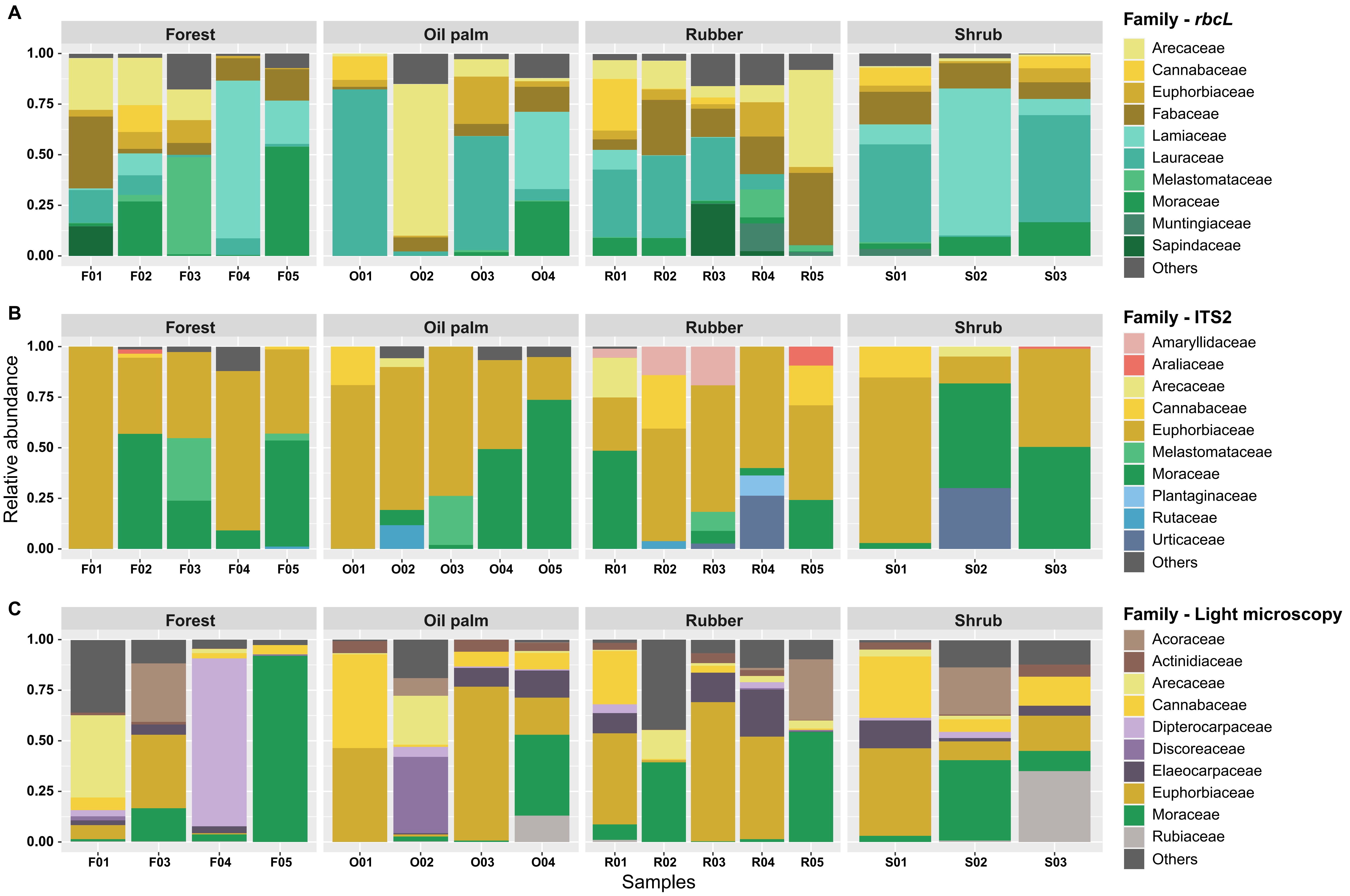

Figure S4. Top 10 plant families detected in pollen samples via (A) DNA metabarcoding – rbcL and (B) ITS2; and (C) light microscopy.
