# Supplementary material for: Biomonitoring via DNA metabarcoding and light microscopy of bee pollen in rainforest transformation landscapes of Sumatra
Source: BMC Ecol Evol. 2022 Apr 26;22:51. doi: 10.1186/s12862-022-02004-x (PMC9040256; doi:10.1186/s12862-022-02004-x)
Supplement: Supplementary file 6 — Additional file 6: Table S1. Sample information of pot-pollen material collected from hives installed in four land-use types (forest, shrub, rubber, and oil palm), including sample IDs used in this study, respective colony IDs, plot coordinates, and percentage of natural cover (pcNatural) within a 500 m of each installed hive. [file 12862_2022_2004_MOESM6_ESM.docx]

**Table S1.** Sample information of pot-pollen material collected from hives installed in four land-use types (forest, shrub, rubber, and oil palm), including sample IDs used in this study, respective colony IDs, plot coordinates, and percentage of natural cover (pcNatural) within a 500 m of each installed hive.

| Sample_ID | Colony_ID | latitude | longitude | pcNatural | Land-use |
| --- | --- | --- | --- | --- | --- |
| F01 | F05A | 1032656 | -190456 | 0,346702 | Forest |
| F01 | F05B | 1032656 | -190456 | 0,346702 | Forest |
| F02 | F09A | 1032988 | -184575 | 0,169482 | Forest |
| F03 | F10A | 1033062 | -182445 | 0,486557 | Forest |
| F04 | F11B | 1033167 | -184556 | 0,170413 | Forest |
| F04 | F11C | 1033167 | -184556 | 0,170413 | Forest |
| F05 | F24A | 1032814 | -179348 | 0,510934 | Forest |
| F05 | F24B | 1032814 | -179348 | 0,510934 | Forest |
| F05 | F24C | 1032814 | -179348 | 0,510934 | Forest |
| S01 | S11A | 1033129 | -183783 | 0,269161 | Shrub |
| S01 | S11B | 1033129 | -183783 | 0,269161 | Shrub |
| S01 | S11C | 1033129 | -183783 | 0,269161 | Shrub |
| S02 | S23A | 1032779 | -179003 | 0,517657 | Shrub |
| S03 | S26B | 1032116 | -178467 | 0,353742 | Shrub |
| S03 | S26C | 1032116 | -178467 | 0,353742 | Shrub |
| R01 | R011A | 1032484 | -19209 | 0,265996 | Rubber |
| R01 | R011B | 1032484 | -19209 | 0,265996 | Rubber |
| R01 | R011C | 1032484 | -19209 | 0,265996 | Rubber |
| R02 | R03A | 1032668 | -191117 | 0,07791 | Rubber |
| R02 | R03B | 1032668 | -191117 | 0,07791 | Rubber |
| R03 | R05A | 1032805 | -186251 | 0,001441 | Rubber |
| R03 | R05B | 1032805 | -186251 | 0,001441 | Rubber |
| R03 | R05C | 1032805 | -186251 | 0,001441 | Rubber |
| R04 | R08C | 1033003 | -185987 | 0,100228 | Rubber |
| R05 | R09A | 1033143 | -184584 | 0,160047 | Rubber |
| O01 | O03B | 1032663 | -190954 | 0,135723 | Oil palm |
| O02 | O10C | 1033122 | -181917 | 0,066864 | Oil palm |
| O03 | O11C | 1033146 | -184734 | 0,106384 | Oil palm |
| O04 | O24B | 1032027 | -180438 | 0,16385 | Oil palm |
| O05 | O25B | 1032027 | -180438 | 0,16385 | Oil palm |
| O05 | O25C | 1032027 | -180438 | 0,16385 | Oil palm |
