# Supplementary material for: Biomonitoring via DNA metabarcoding and light microscopy of bee pollen in rainforest transformation landscapes of Sumatra
Source: BMC Ecol Evol. 2022 Apr 26;22:51. doi: 10.1186/s12862-022-02004-x (PMC9040256; doi:10.1186/s12862-022-02004-x)
Supplement: Supplementary file 10 — Additional file 10: Table S5. Permutational Multivariate Analysis of Variance (PERMANOVA) test based on rbcL data set with the function Adonis (999 permutations) of the Bray-Curtis dissimilarities. [file 12862_2022_2004_MOESM10_ESM.docx]

**Table S5.** Permutational Multivariate Analysis of Variance (PERMANOVA) test based on *rbcL* data set with the function Adonis (999 permutations) of the Bray-Curtis dissimilarities.

| Source of variation | d.f. | Sums of Sqs | Mean Sqs | F.Model | R2 | *P value* |
| --- | --- | --- | --- | --- | --- | --- |
| Land-use type | 3 | 0.8867 | 0.29555 | 0.93091 | 0.17684 | 0.57 |
| Residuals | 13 | 4.1274 | 0.31749 | 0.82316 |  |  |
| Total | 16 | 5.0140 |  | 1.00000 |  |  |
