# Supplementary material for: Biomonitoring via DNA metabarcoding and light microscopy of bee pollen in rainforest transformation landscapes of Sumatra
Source: BMC Ecol Evol. 2022 Apr 26;22:51. doi: 10.1186/s12862-022-02004-x (PMC9040256; doi:10.1186/s12862-022-02004-x)
Supplement: Supplementary file 11 — Additional file 11: Table S6. Permutational Multivariate Analysis of Variance (PERMANOVA) test based on ITS2 data set with the function Adonis (999 permutations) of the Bray-Curtis dissimilarities. [file 12862_2022_2004_MOESM11_ESM.docx]

**Table S6.** Permutational Multivariate Analysis of Variance (PERMANOVA) test based on ITS2 data set with the function Adonis (999 permutations) of the Bray-Curtis dissimilarities.

| Source of variation | d.f. | Sums of Sqs | Mean Sqs | F.Model | R2 | *P value* |
| --- | --- | --- | --- | --- | --- | --- |
| Land-use type | 3 | 0.4424 | 0.14747 | 0.7599 | 0.14003 | 0.68 |
| Residuals | 14 | 2.7168 | 0.19406 |  | 0.85997 |  |
| Total | 17 | 3.1592 |  |  | 1.00000 |  |
