# Supplementary material for: Biomonitoring via DNA metabarcoding and light microscopy of bee pollen in rainforest transformation landscapes of Sumatra
Source: BMC Ecol Evol. 2022 Apr 26;22:51. doi: 10.1186/s12862-022-02004-x (PMC9040256; doi:10.1186/s12862-022-02004-x)
Supplement: Supplementary file 12 — Additional file 12: Table S7. Permutational Multivariate Analysis of Variance (PERMANOVA) test based on light microscopy data set with the function Adonis (999 permutations) of the Bray-Curtis dissimilarities. [file 12862_2022_2004_MOESM12_ESM.docx]

**Table S7.** Permutational Multivariate Analysis of Variance (PERMANOVA) test based on light microscopy data set with the function Adonis (999 permutations) of the Bray-Curtis dissimilarities.

| Source of variation | d.f. | Sums of Sqs | Mean Sqs | F.Model | R2 | *P value* |
| --- | --- | --- | --- | --- | --- | --- |
| Land-use type | 3 | 0.6540 | 0.21799 | 0.68961 | 0.14705 | 0.821 |
| Residuals | 12 | 3.7933 | 0.31611 |  | 0.85295 |  |
| Total | 15 | 4.4473 |  |  | 1.00000 |  |
