# Supplementary material for: Biomonitoring via DNA metabarcoding and light microscopy of bee pollen in rainforest transformation landscapes of Sumatra
Source: BMC Ecol Evol. 2022 Apr 26;22:51. doi: 10.1186/s12862-022-02004-x (PMC9040256; doi:10.1186/s12862-022-02004-x)
Supplement: Supplementary file 13 — Additional file 13: Table S8. Results from One-way ANOVA for the observed richness across the four land-use types (forest, oil palm, shrub, and rubber) detected by ITS2, rbcL, both loci merged and light microscopy. [file 12862_2022_2004_MOESM13_ESM.docx]

**Table S8.** Results from One-way ANOVA for the observed richness across the four land-use types (forest, oil palm, shrub, and rubber) detected by ITS2, *rbcL*, both loci merged and light microscopy.

|  | Observed richness | Df | Sum Sq | Mean Sq | F value | Pr(>F) |
| --- | --- | --- | --- | --- | --- | --- |
| ITS2 | Land-use types  Residuals | 3 | 3.91 | 1.304 | 0.264 | 0.85 |
|  |  | 14 | 69.20 | 4.943 |  |  |
| *rbcL* | Land-use types  Residuals | 3 | 83.1 | 27.68 | 0.99 | 0.428 |
|  |  | 13 | 363.4 | 27.95 |  |  |
| Dual-locus metabarcoding | Land-use types  Residuals | 3 | 162.7 | 54.24 | 1.156 | 0.361 |
|  |  | 14 | 657.1 | 46.93 |  |  |
| Light microscopy | Land-use types  Residuals | 3 | 30.5 | 10.18 | 0.278 | 0.84 |
|  |  | 12 | 439.2 | 36.60 |  |  |
|  |  |  |  |  |  |  |
|  | Shannon | Df | Sum Sq | Mean Sq | F value | Pr(>F) |
| ITS2 | Land-use types  Residuals | 3 | 0.0771 | 0.02571 | 0.288 | 0.833 |
|  |  | 14 | 1.2475 | 0.08911 |  |  |
| *rbcL* | Land-use types  Residuals | 3 | 0.8981 | 0.2994 | 1.524 | 0.255 |
|  |  | 14 | 2.5532 | 0.1964 |  |  |
| Dual-locus metabarcoding | Land-use types  Residuals | 3 | 0.8579 | 0.2860 | 2.45 | 0.107 |
|  |  | 14 | 1.6342 | 0.1167 |  |  |
| Light microscopy | Land-use types  Residuals | 3 | 0.403 | 0.1345 | 0.449 | 0.723 |
|  |  | 12 | 3.597 | 0.2998 |  |  |
|  |  |  |  |  |  |  |
|  | InvSimpson | Df | Sum Sq | Mean Sq | F value | Pr(>F) |
| ITS2 | Land-use types  Residuals | 3 | 2.474 | 0.8245 | 0.998 | 0.422 |
|  |  | 14 | 11.563 | 0.8259 |  |  |
| *rbcL* | Land-use types  Residuals | 3 | 18.89 | 6.296 | 2.163 | 0.141 |
|  |  | 14 | 37.83 | 2.910 |  |  |
| Dual-locus metabarcoding | Land-use types  Residuals | 3 | 38.93 | 12.98 | 2.755 | 0.0816 |
|  |  | 14 | 65.94 | 4.71 |  |  |
| Light microscopy | Land-use types  Residuals | 3 | 3.416 | 1.139 | 0.562 | 0.65 |
|  |  | 12 | 24.320 | 2.027 |  |  |
|  |  |  |  |  |  |  |
