# Supplementary material for: Biomonitoring via DNA metabarcoding and light microscopy of bee pollen in rainforest transformation landscapes of Sumatra
Source: BMC Ecol Evol. 2022 Apr 26;22:51. doi: 10.1186/s12862-022-02004-x (PMC9040256; doi:10.1186/s12862-022-02004-x)
Supplement: Supplementary file 14 — Additional file 14: Table S9. Results of Normality test (Shapiro-Wilk) and Homoscedasticity (Levene’s Test) for alpha-diversity metrics (Observed Richness, Shannon and InvSimpson) estimated for ITS2, rbcL, the merged loci and light microscopy. [file 12862_2022_2004_MOESM14_ESM.docx]

**Table S9.** Results of Normality test (Shapiro-Wilk) and Homoscedasticity (Levene’s Test) for alpha-diversity metrics (Observed Richness, Shannon and InvSimpson) estimated for ITS2, *rbcL*, the merged loci and light microscopy**.**

|  |  | Shapiro-Wilk test | | Levene’s Test | |
| --- | --- | --- | --- | --- | --- |
| Dataset | Alpha-diversity metrics | W | p-value | F-value | Pr(>F) |
| ITS2 | Observed Richness | 0.91687 | 0.1138 | 1.2816 | 0.3191 |
|  | Shannon | 0.79708 | 0.001382 | 0.2394 | 0.8674 |
|  | InvSimpson | 0.96363 | 0.673 | 0.2555 | 0.8562 |
|  |  |  |  |  |  |
| *rbcL* | Observed Richness | 0.98115 | 0.9668 | 2.0618 | 0.1549 |
|  | Shannon | 0.96463 | 0.7194 | 0.1736 | 0.9123 |
|  | InvSimpson | 0.91693 | 0.131 | 0.2468 | 0.8622 |
|  |  |  |  |  |  |
| Dual-locus metabarcoding | Observed Richness | 0.96465 | 0.6931 | 2.2303 | 0.1298 |
|  | Shannon | 0.9664 | 0.7279 | 0.1347 | 0.9377 |
|  | InvSimpson | 0.92912 | 0.1874 | 0.287 | 0.834 |
|  |  |  |  |  |  |
| Light microscopy | Observed Richness | 0.9454 | 0.4205 | 2.5488 | 0.1048 |
|  | Shannon | 0.94923 | 0.4776 | 7.8547 | 0.003647 ** |
|  | InvSimpson | 0.9409 | 0.3602 | 9.4338 | 0.001763** |
