# Supplementary material for: Biomonitoring via DNA metabarcoding and light microscopy of bee pollen in rainforest transformation landscapes of Sumatra
Source: BMC Ecol Evol. 2022 Apr 26;22:51. doi: 10.1186/s12862-022-02004-x (PMC9040256; doi:10.1186/s12862-022-02004-x)
Supplement: Supplementary file 5 — Additional file 5: Figure S5. Workflow of experiment design, laboratory experiments, and summary of pollen metabarcoding pipeline. [file 12862_2022_2004_MOESM5_ESM.pdf]

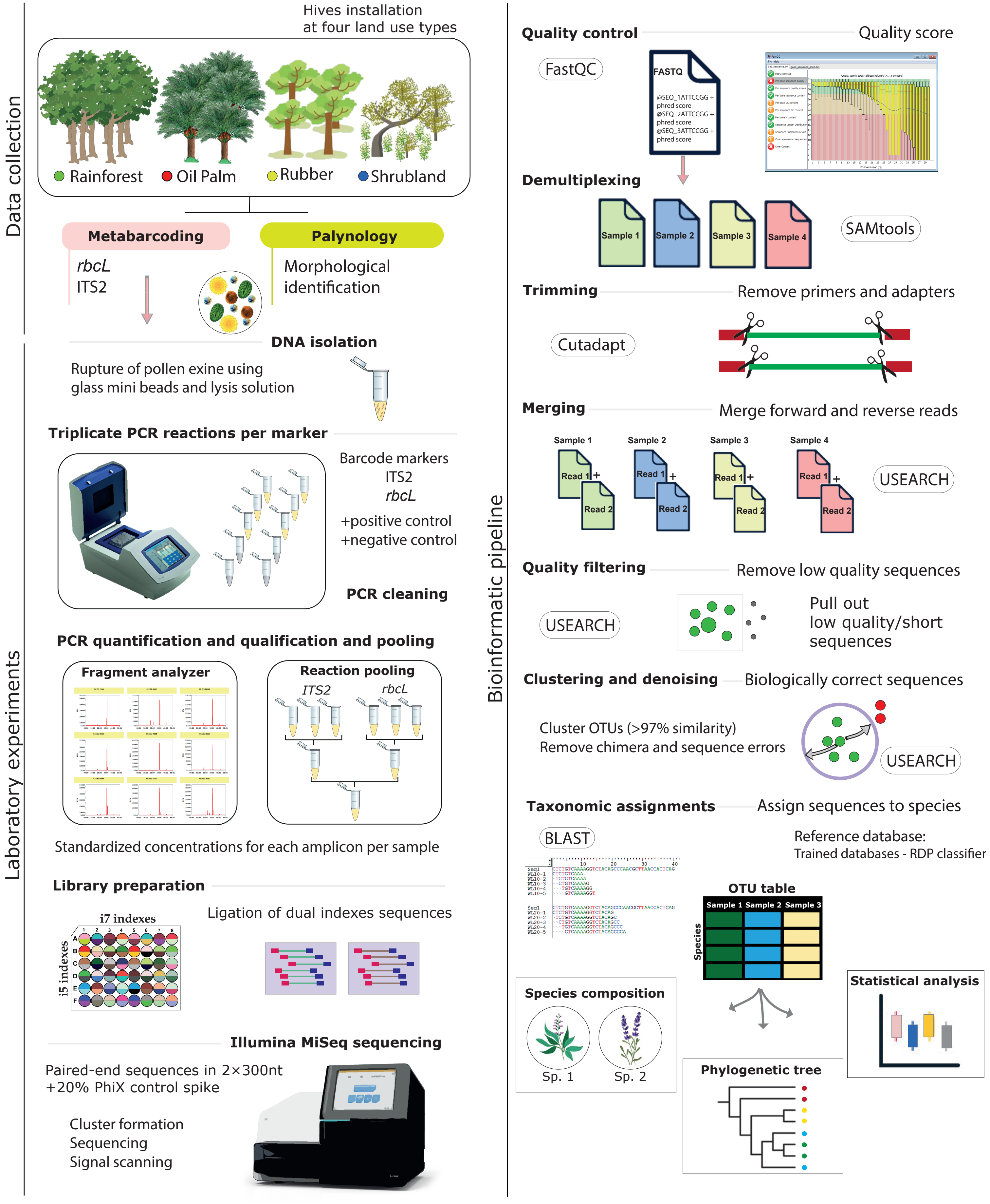

Figure S5. Workflow of experiment design, laboratory experiments, and summary of pollen metabarcoding pipeline.
